# Consistent differences in fitness traits across multiple generations of Olympia oysters

**DOI:** 10.1038/s41598-018-24455-3

**Published:** 2018-04-17

**Authors:** Katherine E. Silliman, Tynan K. Bowyer, Steven B. Roberts

**Affiliations:** 1 University of Chicago, Ecology and Evolution, Chicago, 60637 United States; 2 University of Washington, School of Aquatic and Fishery Sciences, Seattle, 98195 United States

## Abstract

Adaptive evolution and plasticity are two mechanisms that facilitate phenotypic differences between populations living in different environments. Understanding which mechanism underlies variation in fitness-related traits is a crucial step in designing conservation and restoration management strategies for taxa at risk from anthropogenic stressors. Olympia oysters (*Ostrea lurida*) have received considerable attention with regard to restoration, however there is limited information on adaptive population structure. Using oysters raised under common conditions for up to two generations (F1s and F2s), we tested for evidence of divergence in reproduction, larval growth, and juvenile growth among three populations in Puget Sound, Washington. We found that the population with the fastest growth rate also exhibited delayed and reduced reproductive activity, indicating a potential adaptive trade-off. Our results corroborate and extend upon a previous reciprocal transplant study on F1 oysters from the same populations, indicating that variation in growth rate and differences in reproductive timing are consistent across both natural and laboratory environments and have a strongly heritable component that cannot be entirely attributed to plasticity.

## Introduction

Natural environments exhibit spatial heterogeneity in both abiotic and biotic factors, oftentimes driving populations to evolve traits that confer a fitness advantage in their native habitat over foreign genotypes^1^. This process of local adaptation can be opposed by homogenizing gene flow from dispersal, a significant factor for marine species with an extended planktonic dispersal phase^2^. However, if the scale of dispersal extends across strong selective gradients, adaptive population divergence can still occur through phenotype-environment mismatch— where strong purifying selection occurs each generation following dispersal^3,4^. Characterizing the spatial scale and magnitude of adaptive divergence is a crucial step in designing conservation and restoration management strategies for taxa at risk from anthropogenic stressors^5,6^.

Conclusively demonstrating adaptive divergence is complicated by phenotypic plasticity, where individuals adjust their phenotype according to the conditions they experience^7^, which may confound inferences of local adaptation^1,8^. Phenotypic plasticity is widespread in marine species^9–11^, and for marine invertebrates the most common trigger for plasticity appears to be the abiotic environment^11^. Organisms can be raised their entire lives in common conditions in order to minimize the effects of phenotypic plasticity. However this approach may fail with strong transgenerational plasticity (TGP) - defined here as when the environment or phenotype of the parent affects the phenotype of the offspring^1,12^. The ideal experimental design to distinguish TGP from genetic change involves raising and breeding individuals for at least two generations in a common setting^1^, although TGP has persisted for more than two generations in some laboratory studies^13^. Whenever breeding organisms for multiple generations, care should be taken to evaluate and reduce the influences of artificial selection and genetic drift^14^. In a recent review of experimental evidence for local adaptation in marine invertebrates, only 11 out of 59 studies utilized 2 or more generations^15^. Distinguishing between plastic responses and adaptive evolution to the environment is key to understanding the potential for marine species to acclimate or adapt to changing environmental conditions^16^.

Marine molluscs, and bivalves in particular, constitute some of our most economically and ecologically important marine invertebrates. Like many other marine invertebrates, they exhibit complex life cycles which include both planktonic larval stages as well as benthic juvenile and adult stages. The larval stage of many marine molluscs has been shown to be particularly sensitive to risks from ocean acidification and warming^17,18^, resulting in an increasing need to understand the relative importance of adaptive and plastic processes in shaping phenotypic variation. Evidence that TGP might be common for marine molluscs is growing (see ref.^19^ for a thorough review), however only a handful of studies investigating adaptive differentiation in this clade have compared organisms raised in common conditions for at least 2 generations, and of those most involved Gastropoda (see refs^20–24^).

The Olympia oyster (*Ostrea lurida*) is an estuarine species natively distributed from the central coast of Canada to Baja California. As a rhythmic consecutive hermaphrodite, spawning events in *O*. *lurida* are thought to be synchronized between males and females based on environmental cues of temperature and seasonality^25^. Considered ecosystem engineers in estuaries, they provide structured habitat, remove suspended sediments, and limit eutrophication^26^. Following devastating commercial exploitation in the early 20th century, recovery of Olympia oyster populations has been stifled by other anthropogenic threats (water quality issues, habitat loss, and possibly ocean acidification)^27,28^. There is political and economic pressure to restore abundance and recover ecosystem services offered by this species, which has spurred increasing interest in understanding the genetic, phenotypic, and epigenetic variation at both local and regional scales^29^.

A recent study on Olympia oysters in central California provided evidence for spatial adaptive differentiation through a reciprocal transplant experiment with first-generation laboratory-reared (F1) oysters. The authors also found suggestive, although not statistically significant, evidence of population-level differences in low salinity tolerance in second-generation, laboratory-reared (F2) oysters, and hypothesized adaptive divergence may occur in Olympia oysters over distances as short as 20–100 km^24^. In Puget Sound, WA, Heare *et al*.^30^ conducted a reciprocal transplant experiment with F1 Olympia oysters from three distinct populations (Dabob Bay, Oyster Bay, and Fidalgo Bay) in 2014. Variation in survival, growth rate, and reproductive activity was observed among populations and the four transplant sites. In particular, oysters from Fidalgo Bay had faster growth rates and reduced or delayed reproductive activity at most sites, while oysters from Dabob Bay had better survival yet slower growth rates, indicating potential adaptive trade-offs^30^.

Although both of these studies controlled for environmental exposure of broodstock for up to 5 months prior to producing F1 oysters and attempted to maximize genetic diversity, they did not sufficiently minimize concerns about TGP or evaluate the relatedness of their laboratory-reared populations. This study aims to mitigate the influence of TGP on inferring adaptive differentiation by testing if phenotypic differences among populations of Olympia oysters are consistent across generations. In the summer of 2015, we conducted a common garden experiment on F1 and F2 oysters derived from the same three Puget Sound populations as Heare *et al*.^30^. Three fitness-related traits were measured across populations- reproductive activity, larval growth rate, and juvenile growth rate. We also estimated the relatedness and genetic diversity of the F1 generation using SNP data in order to demonstrate that the population-specific traits described here are not primarily due to family-level variation from few effective breeders.

## Results

### Reproductive Activity

**Figure 1:**
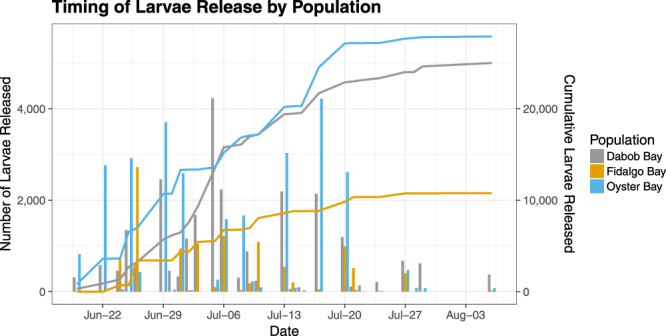
Reproductive activity in F1 oysters from three Puget Sound populations. The left axis measures the number of larvae released on each sampling day, summed across replicates. The right axis shows the cumulative number of larvae produced through time. Both measures are normalized by the number of adult oysters in each population.

**Figure 2:**
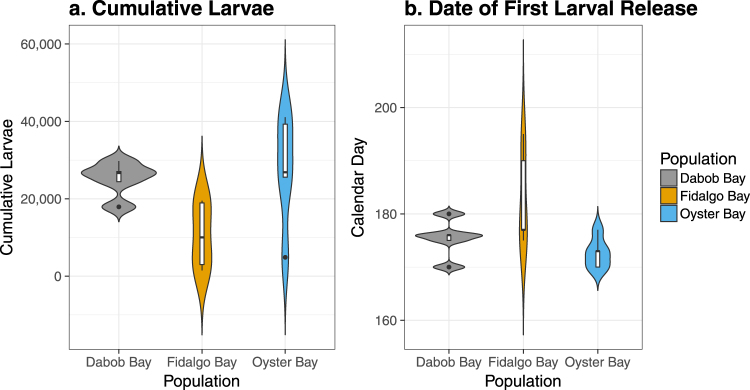
(**a**) Cumulative number of larvae released within each replicate bucket over 7 weeks, normalized by the number of oysters in each bucket. Oysters from Fidalgo produced significantly fewer larvae than those from Oyster Bay (Tukey post hoc test, *p* = 0.0499) and fewer larvae, although not significantly, than those from Dabob (Tukey post hoc test, *p* = 0.0954). (**b**) Calendar day of first observed larvae release. Fidalgo oysters released larvae 10 days later than Oyster Bay oysters (Tukey post hoc test, *p* = 0.0434) and 7 days later than Dabob oysters on average, although this was not statistically significant (Tukey post hoc test, *p* = 0.156).

The timing and quantity of larvae produced varied across the three populations (Fig. 1). The cumulative number of larvae produced over a 7 week period differed significantly among population (one-way ANOVA, Df = 2, F = 4.174, *p* = 0.0421), with F1 oysters from Fidalgo producing the fewest (Fig. 2a). Combined across all replicates, Oyster Bay oysters produced 2.7 million larvae, Dabob oysters produced 2.4 million, and Fidalgo oysters produced 1.1 million.

The onset of larval release also differed significantly among populations (one-way ANOVA, Df = 2, F = 4.033, *p* = 0.0457), with Fidalgo oysters exhibiting delayed reproduction compared to the other populations and much higher variance in the date of initial larval release (Fig. 2b). The timing of peak larval production did not vary significantly among populations (ANOVA, Df = 2, F = 0.097, *p* = 0.908).

### Larval Growth Experiment

**Figure 3:**
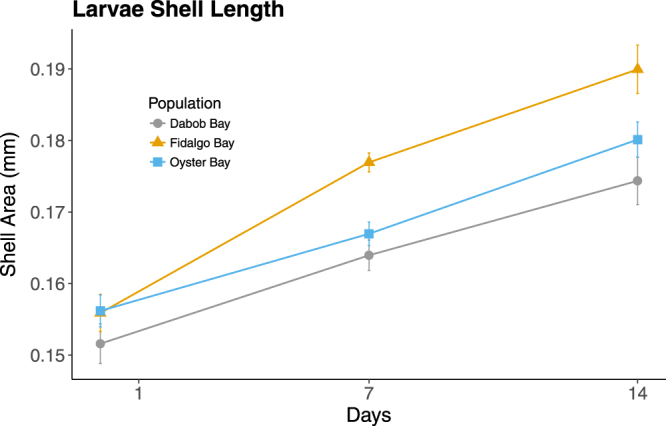
Larval shell length of F2 oysters from three populations over 14 days. Data are means across replicates += s.e.m. Size varied significantly among populations at Day 7 (LMM, *p* = 7.939e^−5^) and Day 14 (LMM, *p* = 0.03573).

Significant differences in larvae size were not detected among populations on Day 0 of the larval growth experiment (one-way ANOVA, Df = 2, F = 0.939, *p* = 0.401). By Day 7, size varied significantly among populations (linear mixed model(LMM), *p* = 7.939e^−5^). Fidalgo larvae were 8% larger than Dabob larvae (t-test, *p* = 1.8e^−6^) and 6% larger than Oyster Bay larvae (t-test, *p* = 0.00026). After 14 days, size still varied significantly among populations (LMM, *p* = 0.03573), but only the comparison between Fidalgo and Dabob larvae remained significant (9% larger; t-test, *p* = 0.0017) (Fig. 3).

### Juvenile Growth Experiment

**Figure 4:**
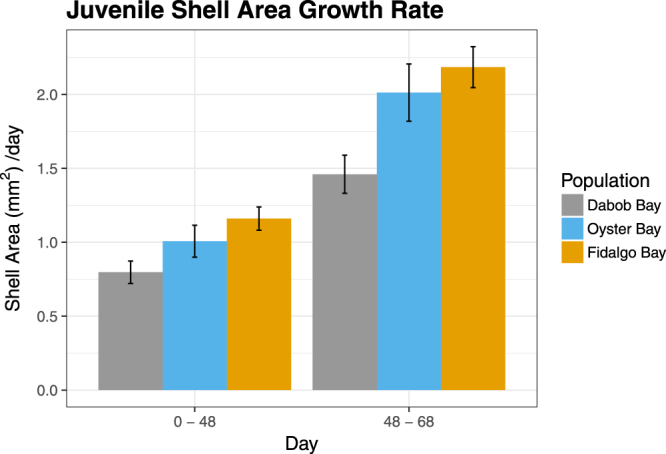
Juvenile shell area growth rate of F2 oysters from three populations over 9 weeks. Juvenile shell growth of F2 oysters (growth rate = Δarea/# days). Growth rate between Day 0 - Day 48 differed significantly among populations (LMM, *p* = 0.02236) as well as between Day 48 - Day 68 (LMM, *p* = 0.0012).

Significant differences in juvenile shell area at Day 0 were not detected (one-way ANOVA, Df = 2, F = 0.483, *p* = 0.617). Growth rate between Day 0 and Day 48 diverged among populations (LMM, *p* = 0.02236). Fidalgo oysters grew 46% faster than Dabob oysters (Kruskal-Wallis post hoc test, *p* = 0.011), but all other pairwise comparisons were not significant. Between 48 and 68 days, shell growth continued to differ among populations (LMM, *p* = 0.0012). Dabob oysters grew slower over this time period than Fidalgo oysters (Kruskal-Wallis post hoc test, *p* = 0.0027) and tended to grow slower than Oyster Bay oysters (Kruskal-Wallis post hoc test, *p* = 0.0806) (Fig. 4).

### Genetic Diversity and Relatedness

Population-level estimates of genetic diversity and relatedness for F1 oysters are shown in Table 1. Observed heterozygosity (*H*_*o*_), expected heterozygosity (*H*_*e*_) and inbreeding coefficient (*F*_*is*_) represent values averaged over loci that are polymorphic within the population, while kinship coefficient (*ϕ*) is averaged over all pairwise comparisons of individuals. Values of *ϕ* over 0.1768 are considered full-sibs. Observed heterozygosity varied across loci and did not significantly differ from expected heterozygosity except in Dabob oysters (Bartlett’s test, *p* = 0.02726). Sibship reconstruction identified families of full-sib pairs within the 50 oysters sampled from each population. Fidalgo oysters comprised of 33 families (including “families” with a single individual), none of which included more than 8% of the total individuals. Dabob oysters had 21 full-sib families and Oyster Bay oysters had 17 families, the largest of which contained 16% and 24% of individuals, respectively. Families for all populations were well mixed between spawning groups for production of F2 oysters.

**Table 1:** *H*_*o*_, observed heterozygosity; *H*_*e*_, expected heterozygosity; *F*_*is*_, inbreeding coefficient; *ϕ*, kinship coefficient.

	Fidalgo Bay	Dabob Bay	Oyster Bay
*H*_*o*_	0.1957	0.2000	0.2151
*H*_*e*_	0.2081	0.2064	0.2227
*F*_*is*_	0.0593	0.0309	0.0341
*ϕ*	0.0191	0.0436	0.0372

## Discussion

The results presented here provide evidence for a strong heritable component underlying phenotypic variation in growth and reproduction among three populations of Olympia oysters in Puget Sound, WA. By building on the Heare *et al*.^30^ study and showing consistent interpopulation differences between first-generation (F1) and second-generation (F2), commonly reared oysters, this study is the first to demonstrate statistically significant phenotypic population structure extending through the F2 generation in *Ostrea lurida*, and one of the few to do so in a mollusc. Persistent population differences in growth rate and reproductive timing across several generations have also been documented for selectively-bred strains of the eastern oyster, *Crassostrea virginica*^23,31^, suggesting population divergence at these traits may be general across Ostreidae.

Reproductive activity was characterized for the adult F1 oysters, with Oyster Bay oysters producing the most larvae over the course of 7 weeks and the Fidalgo population demonstrating delayed reproductive activity, in accord with the reciprocal transplant study by Heare *et al*. By showing consistent reproduction patterns in both variable natural environments and controlled laboratory conditions, we demonstrated that reproductive timing in this species is not exclusively mediated by environmental conditions, but is also under genetic and/or epigenetic control. This result is in concordance with recent studies indicating that nearby populations of Olympia oysters vary in temperature thresholds for reproduction^32,33^. The development of asynchronous reproduction on such small spatial scales has major implications for limiting gene flow and contributing to population divergence^34^.

Fidalgo oysters exhibited the fastest growth during both the F2 larval and juvenile stages, while Dabob oysters exhibited the slowest growth, resulting in a significantly smaller size than Fidalgo oysters and smaller (although not significantly) than Oyster Bay oysters. This result is consistent with findings in F1 oysters by Heare *et al*., indicating a fixed underpinning for growth rate differences that manifests during both the larval and juvenile life stages. Interestingly, Fidalgo oysters also exhibited severely reduced and delayed reproductive activity. One explanation for this is an adaptive trade-off in energy allocation^35,36^, where Fidalgo oysters are devoting more energy to shell growth and less to gonad development. Heare *et al*. also observed a potential adaptive trade-off in their reciprocal transplant study, where the population with slowest growth also had the highest survival across sites. Further investigation is required to fully resolve the link between growth, reproductive activity, and survival.

Given the characteristically high variance in reproductive success for *Ostrea* species^37,38^, we estimated genetic diversity and relatedness for a subset of F1 oysters to ensure our approach of separating broodstock into smaller groups of 20–25 oysters produces offspring with many different families represented. Assuming the original wild population had kinship coefficients close to zero, our results do indicate that a reduced number of the original broodstock contributed to the F1 generation. However, by identifying between 17 and 33 different families within 50 sampled individuals, we are confident that the total pool of 94–101 F1 individuals per population represents upwards of 40 wild genotypes and that our spawning method of mixing these families across smaller groups produced a genetically diverse F2 generation.

Environmental conditions in temperature, freshwater input, primary productivity, and anthropogenic waste effluent are known to vary among these sites^30^, supporting the possibility of selection driving the phenotypic variation observed in this study. In particular, up to 10 fold less chlorophyll *a* has been observed in Fidalgo Bay compared to the other sites. The proposed trade-off in energy allocation for this population could be driven by selection from such environmental differences in the quantity and timing of food supply^39,40^. As mentioned previously, selection could act through local adaptation or phenotype-environment mismatch, depending on the spatial scale of dispersal relative to the scale of environmental heterogeneity. Although dispersal information is not currently available for Olympia oyster larvae in Puget Sound, estimates using chemical fingerprinting in Southern California identified considerable larval exchange among estuaries separated by up to 75 km^41^, which encompasses the distance between the populations used in this study. Further research using both chemical fingerprinting and genetic approaches are required to understand patterns of dispersal within Puget Sound in order to elucidate the mechanisms of adaptive population divergence.

Our results have implications for ongoing restoration efforts attempting to rebuild Olympia oyster populations. Current protocols for hatchery-based Olympia oyster restoration in Puget Sound involve using wild broodstock to produce hatchery-raised juveniles for outplanting in the same source population as the broodstock^27^. These efforts have focused on multiple sites in Puget Sound, including those covered in this study. Our results support this practice, with a couple of caveats: (1) If local adaptation is indeed driving the observed phenotypic population structure, populations may be adapted to historical, rather than current, conditions. The convention of replenishing populations with only local broodstock sources may not provide the genetic diversity required to adapt in the face of rapid anthropogenic-induced environmental changes^42^. (2) Hatchery conditions vary dramatically from natural summer spawning conditions, and therefore artificial selective pressures are likely at play in the production of both the F1 and F2 oysters used in this study^14^. However, the observed differences in size among populations are corroborated by field observations of wild oysters (Brady Blake, WA Dept of Fish and Wildlife, 7-10-2017, pers comm).

Despite the relatively close proximity of these populations and the potential for high gene flow, we observed significant phenotypic differences in fitness-related traits, even after multiple generations in the same environment. Of note, the population with the fastest growth rate also exhibited delayed and reduced reproductive activity, indicating a potential adaptive trade-off. By mitigating the influence of transgenerational plasticity, our results suggest these trait differences are heritable. Further research is now required to understand the mechanisms of inheritance underlying these observed differences, whether they be genetic or epigenetic.

## Methods

### Broodstock

Adult oysters were collected from three locations in Puget Sound, Washington; Fidalgo Bay (N 48.478252, W 122.574845), Oyster Bay (N 47.131465, W 123.021450), Dabob Bay (N 47.850948, W 122.805694) during November and December 2012. Oysters were held for 5 months in common conditions in Port Gamble, Washington and spawned in June 2013. Unlike many other oyster genera that broadcast spawn both eggs and sperm (e.g. *Crassostrea*), Olympia oyster females are fertilized with spermatozeugmata (‘sperm packets’) from the water column and brood larvae for approximately 10–12 days. After being released into the water column, larvae are planktonic for approximately two weeks before attaching to a hard substrate (‘settlement’). To ensure genetic diversity, each population was allowed to spawn in 24 separate groups of 20–25 oysters. Larvae produced from each population were reared in tanks based on spawning group, settled on very small pieces of oyster shell, then fed ad libitum. In August 2013, 480 juvenile oysters (5–10 mm) from each source population were outplanted at Clam Bay located in central Puget Sound (N 47.571839, W 122.550813), a different site than any of the source populations. For the purposes of this study we will refer to the cohort outplanted at Clam Bay as F1s. Reproductive and growth characteristics of F1 oysters at Clam Bay have been described by Heare *et al*.^30^. In June 2015, F1 oysters were moved into NOAA’s Kenneth K. Chew Center for Shellfish Research and Restoration in Manchester, WA and maintained in mesh bags suspended in separate 18.9 L buckets with a diet of mixed live algae in flowing seawater.

### Larval Rearing and Quantifying Reproductive Activity

Spawning of broodstock (F1) was induced by elevating temperature to 18–20 °C in May 2015 and maintaining algae supplementation at 60,000–80,000 cells/mL. To ensure genetic diversity, each population (Fidalgo Bay = 101, Oyster Bay = 100, Dabob Bay = 94) was divided into 5 groups of 16–21 oysters. In addition to ensuring that multiple females were involved in reproduction, these replicate groups allowed us to statistically test population-level differences in reproductive activity. Larval release was checked and quantified every one to three days with larvae filtered (100 *μ*M) and counted with triplicate drop counts. A cohort of these F2 larvae were used in the larval growth trials with the remainder raised in tanks (100 L) for the juvenile growth trial.

### Experimental Set-up: Larvae Growth

To investigate differences in growth rate among populations at the larval stage, we set up a larval growth rate experiment starting on a day when all three populations were producing at least 25,000 total larvae across multiple buckets (i.e. multiple females). Larvae were pooled by population and mixed well in 1 L beakers. The concentration of larvae was estimated using triplicate drop counts, and 900 larvae per population were added to each of three replicate plastic beakers (1 L) in order to account for random effects due to the beaker environment. These beakers were each outfitted with a “silo”, a 7.62 cm (3”) diameter section of conditioned PVC pipe covered in 100 *μ*M mesh on the bottom. Larvae remained in the silo, which allowed for daily water changes by lifting up the silo and inserting into a new beaker with premixed fresh seawater (800 ml) containing a 50/50 mix of live *T*. *isochrysis* and diatom algae (final concentration 60,000–80,000 cells/mL). Approximately 20 larvae were sampled haphazardly by pipette from the initial larval pools at Day 0 and each replicate at Day 7 and Day 14 of the experiment, fixed in 10% buffered formalin, and photographed under a microscope for analysis using ImageJ v1.51^43^ software to determine shell area and length. We report results based on shell length, although shell area gave qualitatively similar results (not shown).

### Experimental Set-up: Juvenile Growth

Larvae from each population greater than 224 *μ*M (n = 30,000) were moved to new tanks (100 L) where air was bubbled to maintain oxygen levels and stimulate water movement. These tanks were lined with PVC tiles (10 × 10 cm) roughed on one side using coarse sandpaper to promote oyster settlement. After four weeks, settled oysters were culled to fewer than 30 oysters/tile to avoid overgrowth interactions (Fidalgo tiles = 10, Oyster Bay tiles = 7, Dabob tiles = 8). Tiles were randomized and attached to four protected outplanting trays that were suspended from the dock at NOAA Manchester Research Station (depth = 6 m). Photos were taken of oysters on tiles prior to outplanting (Day 0), after 48 days, and after 68 days for analysis using ImageJ software to determine shell area.

### 2b-RAD Genotyping

Using a 2b-RAD reduced-representation sequencing approach^44^, we sequenced 279 individuals and 18 technical replicates from the F1 generation for a total of 297 samples across 5 lanes of Illumina HiSeq. The technical replicates are necessary for quality assessment and genotyping recalibration of downstream analyses. For each population, we mapped reads from the 50 individuals with the highest read depth as well as 4–5 technical replicates to a draft *O*. *lurida* genome of 8,733 scaffolds over 10 kb in length. For each sample in this subset, approximately 30% of reads mapped to the draft genome using Bowtie2. After genotyping single nucleotide polymorphisms (SNPs) using the UnifiedGenotyper tool in GATK v3.6^45^, we filtered SNPs for quality and excess heterozygosity based on Hardy-Weinberg equilibrium within populations using VCFtools^46^ and custom scripts by Mikhail Matz https://githb.com/z0on/2bRADGATK. For subsequent analyses of relatedness and genetic diversity, we thinned our dataset to one SNP per 2b-RAD tag for 677 SNPs confidently identified in at least 75% of individuals.

### Analysis

To compare differences in reproductive activity among populations in the F1 generation, the five separate broodstock buckets per population were considered as independent replicates. The number of larvae released in each bucket on each sampling day was normalized by the number of adults in that bucket. To determine if there was a difference in total reproductive output among populations, the cumulative number of larvae produced throughout the 7 weeks for each bucket was analyzed using a one-way analysis of variance (ANOVA; R base) with post hoc Tukey’s tests. In order to determine if populations differed in their timing of reproductive activity, one-way ANOVAs were also conducted on the number of days until the first observed larval release in each bucket after spawning conditions were induced, as well as the number of days until spawning peaked in each bucket.

Linear mixed models (LMMs) were used to measure the effect of source population on oyster growth using the R package *lme4*^47^. For the larval growth experiment, randomly selected live larvae were measured on Day 0, Day 7 and Day 14 with 10–12 larvae (mean = 11.8, std = 0.62) larvae per replicate. Dead oysters are easily distinguished from living oysters by having an entirely clear protoshell and no observable tissue. As *O*. *lurida* larvae grow prior to maternal release, a one-way ANOVA was used to test if size varied among populations on Day 0 prior to separating out into replicate beakers. For the LMM analysis, population was a fixed effect and replicate beaker was a random effect. Prior to running the LMM, size distributions were tested for normality using the Shapiro-Wilkes test with the stats R package. Significance of the LMM results was established using a Likelihood Ratio Test against a null model based only on random effects. Shell length was compared at each time point using pairwise t-tests with a Bonferroni correction for multiple testing.

For the juvenile experiment, growth was tracked on an individual basis (growth rate = Δarea/# days). All oysters that were alive (as determined by a healthy shell color and response to prodding) and not extending > 50% off the tile at each time point were measured for shell area. For the linear mixed model, population was a fixed effect and tray containing the tile was a random effect. Growth rate was natural log-transformed based on indications of non-normal distributions from the Shapiro-Wilkes test. Pairwise comparisons for populations at each time point were performed with the Nemenyi post hoc test using information from the Kruskal-Wallis test (*PMCMR* package)^48^.

Estimates of genetic diversity and relatedness in the F1 generation were calculated in R v3.4.1^49^ using 677 high-quality SNPs obtained by 2b-RAD sequencing. Mean expected and observed heterozygosity was calculated for each population using hierfstat^50^ and compared with Bartlett’s test of homogeneity of variances^51^. Pairwise estimates of relatedness were calculated using the KING algorithm^52^ as implemented in VCFtools^46^. Full sib pairs are classified by having a kinship coefficient over $\frac{1}{{2}^{\frac{3}{2}}}$. Sibship was estimated in each population using a full-maximum likelihood model as implemented by Colony v. 2.0.6.4^53,54^, with the following parameters: polygamous males and females, long run length, full-likelihood analysis, high-likelihood precision, update allele frequencies during run, no prior information, and allelic dropout rate of 0.001.

### Data and Code Availability

The datasets generated during current study are available on figshare, 10.6084/m9.figshare.5975452. Reproducible R Markdown notebooks detailing the code used for statistical analyses can be found at www.github.com/ksil91/PS-Oly-Larvae-Growth.
